# A Comprehensive Analysis of Codon Usage Patterns in Blunt Snout Bream (*Megalobrama amblycephala*) Based on RNA-Seq Data

**DOI:** 10.3390/ijms160611996

**Published:** 2015-05-26

**Authors:** Xiaoke Duan, Shaokui Yi, Xianwu Guo, Weimin Wang

**Affiliations:** 1 College of Fisheries, Key Lab of Agricultural Animal Genetics, Breeding and Reproduction of Ministry of Education/Key Lab of Freshwater Animal Breeding, Ministry of Agriculture, Huazhong Agricultural University, Wuhan 430070, China; E-Mails: xiaokeduan@126.com (X.D.); yishaokui@foxmail.com (S.Y.); 2 Lab of Biotecnología Genómica, Centro de Biotecnología Genómica, Instituto de Politécnico Nacional, Boulevard del Maestro S/N esq. Elías Piña, Col. Narciso Mendoza, Tamaulipas 88710, Mexico; E-Mail: xguo@ipn.mx

**Keywords:** RNA-Seq, *Megalobrama amblycephala*, codon usage, codon pairs, GC_3_ biology, methylation site, vertebrate evolution

## Abstract

Blunt snout bream (*Megalobrama amblycephala*) is an important fish species for its delicacy and high economic value in China. Codon usage analysis could be helpful to understand its codon biology, mRNA translation and vertebrate evolution. Based on RNA-Seq data for *M. amblycephala*, high-frequency codons (CUG, AGA, GUG, CAG and GAG), as well as low-frequency ones (NUA and NCG codons) were identified. A total of 724 high-frequency codon pairs were observed. Meanwhile, 14 preferred and 199 avoided neighboring codon pairs were also identified, but bias was almost not shown with one or more intervening codons inserted between the same pairs. Codon usage bias in the regions close to start and stop codons indicated apparent heterogeneity, which even occurs in the flanking nucleotide sequence. Codon usage bias (RSCU and SCUO) was related to GC_3_ (GC content of 3rd nucleotide in codon) bias. Six GO (Gene ontology) categories and the number of methylation targets were influenced by GC_3_. Codon usage patterns comparison among 23 vertebrates showed species specificities by using GC contents, codon usage and codon context analysis. This work provided new insights into fish biology and new information for breeding projects.

## 1. Introduction

Blunt snout bream (*Megalobrama amblycephala* Yih, 1955), naturally distributed in the middle and lower reaches of the Yangtze River in China [1], has been one of the main aquaculture fish in China due to being a delicacy since the 1960s [2]. Due to its high economic value, the total production of *M. amblycephalais* is rapidly growing [3]. In recent years, molecular techniques, deep sequencing for transcriptome and microRNAs analysis were initially used for the development of molecular markers associated with several important economic traits, such as body shape, hypoxia resistance and disease resistance [4,5,6].

Triplet codons are the basic coding units in mRNAs, playing roles in coding for a particular amino acid or causing initiation or termination of a protein translation [7]. Due to the degeneracy of genetic code, synonymous codons are translated into the same amino acid except Met and Trp. Despite synonymous mutations being silent in protein sequences according to the central dogma, synonymous codon bias exists widely within and between genomes [8]. The wide variations in codon usage patterns of many organisms have provided clues to help understand genome evolution and some aspects of molecular biology [7,9,10,11,12].

The study of codon usage based on the full length ORF (open reading frame) sequences or genomes has been documented in a wide variety of organisms such as cyanobacteria [10], *Caenorhabditis*, *Drosophila*, *Arabidopsis* [11], *Silene latifolia* [12] and insects [13]. However, no similar study has been performed with fish. To date, large amounts of sequence data can be obtained through genome sequencing or RNA-Seq, providing an opportunity for the species-specific analysis of codon usage patterns in more non-model organisms. In the present study, using the RNA-Seq data for *M. amblycephala* (Accession No.: SRA045792) [4], codon usage patterns of *M. amblycephala* were revealed by the analysis of codon usage and codon pairs, position-dependent codon usage bias, GC_3_ bias and comparison among the vertebrates. The results will improve our understanding of codon biology in *M. amblycephala* and fish evolution and will have potential applications for fish breeding.

## 2. Results and Discussion

### 2.1. Codon Usage in M. amblycephala

Codon usage analysis in *M. amblycephala* was based on 646 full-length ORF sequences after a filtering series from 100,477 unigenes (contigs and singletons), which were assembled and annotated in our previous work [4].

**Figure 1:**
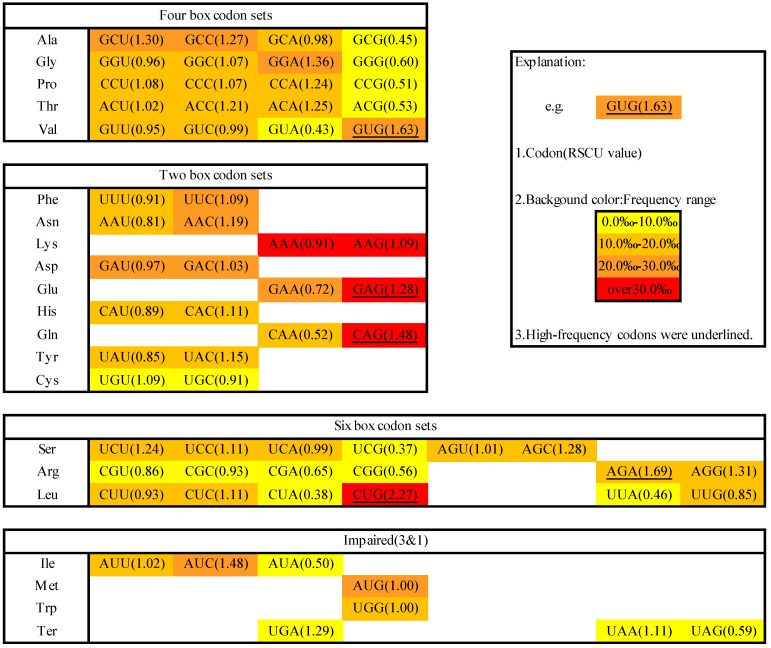
Unequal usage of 64 codons in *M. amblycephala*. The codons for the same amino acids are listed on the left and are colored yellow, orange yellow, orange and red to show occurrence frequencies of 0.0‰–10.0‰, 10.0‰–20.0‰, 20.0‰–30.0‰, and over 30.0‰, respectively. The data is shown as a triplet codon (RSCU, relative synonymous codon usage), and high-frequency codons are underlined.

A codon usage table was created by investigating all 138,002 codons (Table S1 in Supplementary file 1). With each codon, excepting three stop codons, GAG was most frequently presented, with the occurrence of 45.8‰, 2.8 times the average frequency. UCG was the lowest frequency codon (4.4‰) and another 14 codons also had low frequency (<10‰) (Figure 1). Measured by their codon frequencies, abundant and rare codons were defined as the 15 most abundant and 15 most rare codons in *M. amblycephala*. The overall GC content in the study is 0.494, but it varies among different codon positions, with the highest in GC_3_ (GC content of 3rd nucleotide in codons), at a value of 0.558, lowest in GC_2_ (GC content of 2nd nucleotide in codons), at a value of 0.389, and intermediate in GC_1_ (GC content of 1st nucleotide in codons), at a value of 0.534, which is consistent with the observations in other fishes (Table S2).

Based on the occurrence of synonymous codons of all 59 codons (excluding the Met, Trp, and three stop codons), five codons were identified as high-frequency codons (Figure 1). CUG (Leu), AGA (Arg) and GUG (Val) had the highest values (2.27, 1.69 and 1.63, respectively). CAG (Gln) and GAG (Glu) were used much more frequently than other synonymous codons for the corresponding amino acids (74.2% and 63.8%, respectively).

However, four NUA codons in *M. amblycephala* had quite low RSCU (0.46, 0.38, 0.50, and 0.43) (Figure 1). The reduction of UA may increase protein production by means of inhibition of mRNA degradation [14]. Four NCG codons also showed low RSCU value (0.37, 0.51, 0.53 and 0.45). This phenomenon may be conducive to avoiding possible mutation caused by DNA methylation. As the methylated cytosine (C) in the CG dinucleotide is more easily deaminated into thymine (T), and the G in the 3rd codon position is wobbly, species with a high level of DNA methylation tend to avoid NCG codons to produce fewer mutations [15,16]. The low RSCU of NCG codons indicate that *M. amblycephala* may be a species with a relatively high methylation level. Meanwhile, NCG:NCC, a ratio widely used to estimate CpG suppression and to reflect the methylation level in mRNA coding sequences [15,16], was relatively low in *M. amblycephala* (0.394) compared with other fishes (Table S2), also confirming that *M. amblycephala* had a high methylation level.

As for stop codons, both UGA and UAA were the preferred stop codons, with RSCU value of 1.29 and 1.11, respectively, and UAG (0.59) was the least frequently used (Figure 1), concordant with the overall rules discerned for vertebrate animals [17].

### 2.2. Codon Pairs in M. amblycephala

Unequal usage also existed for synonymous codon pairs. Based on the usage of all 3717 synonymous codon pairs (excluding the AUGAUG, AUGUGG, UGGAUG and UGGUGG codon pairs), 724 high-frequency codon pairs were identified by using the SSC (shuffled synonymous codons) null model (Supplementary file 2), among which CUGCUG encoding LeuLeu was found to be the top synonymous codon pair with the RSCPU of 5.82. AGAGGA (ArgGly, 4.55), UCCAGA (SerArg, 4.51), UCCACC (SerThr, 4.28), CUGGCC (LeuAla, 4.20), CAGCUG (GlnLeu, 4.07), GCCAUC (AlaIle, 4.05) ranked as the 2th to 7th highest frequency codon pairs. Only 335 amino acid pairs, rather than all 396 amino acid pairs (excluding the MetMet, MetTrp, TrpMet and TrpTrp amino acid pairs) were encoded by those 724 high-frequency codon pairs, indicating that the majority of amino acid pairs had obvious bias for synonymous codon pairs in *M. amblycephala*.

**Figure 2:**
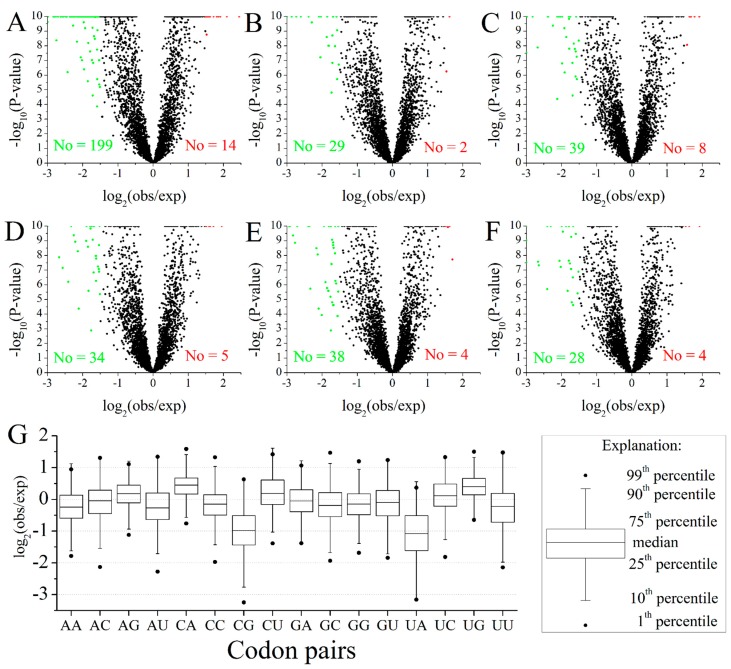
Overview of preferred and avoided codon pairs in *M. amblycephala*. (**A**–**F**) The relationship between observed frequency and expected frequency of 3721 (61 × 61) codon pairs (excluding stop codons). The red, green and black spots represent the preferred codon pairs, avoided codon pairs and unbiased codon pairs, respectively. The lowest *p*-value was set to 1 × 10^−10^. (**A**) The ratio of observed frequency to expected frequency for neighboring codon pairs; The ratio of observed frequency to expected frequency for codon pairs separated by one (**B**); two (**C**); three (**D**); four (**E**) and five (**F**) intervening codons; (**G**) Distribution of the ratio of observed frequency to expected frequency for different neighboring codon pairs. The *X* axis shows the dinucleotide at the junction of neighboring codon pairs.

Based on the observed and expected frequency of all 3721 neighboring codon pairs (61 × 61) without the constraint of synonymous codon pairs, 14 preferred codon pairs and 199 avoided codon pairs were observed by using SC (shuffled codons) null model (Figure 2A). The number of avoided codon pairs was much larger than preferred codon pairs suggesting that the selection acts primarily through avoidance of the most disadvantageous codon pairs [18]. However the bias nearly disappeared when one to five intervening codons inserted between the pairs were tested (Figure 2B–F, Supplementary file 2). This phenomenon is consistent with the mechanism that neighboring codon pair bias may have significance in protein synthesis [19].

Among the 14 preferred neighboring codon pairs, the top three presented patterns were nnCAnn (21.4%), nnUGnn (21.4%) (Figure 2G) and simple codon repeats (GCGGCG, CCGCCG) (Supplementary file 2), which were a little different from the studies in bacteria, archaea and other eukaryotes [7,18]. The type of simple codon repeats may play an important role in slowing down the translation rate of the corresponding mRNAs [7]. As a former tRNA was still linked to mRNA, leading to a decrease of concentration of the same tRNA in free state in a cell, it would take more time for mRNA to obtain the same tRNA. Of 199 avoided neighboring codon pairs, 75 pairs (37.7%) had UA at the junction and 56 pairs (28.1%) showed a nnCGnn pattern (Supplementary file 2), which were consistent with the low frequency of NUA and NCG codons and may play similar roles in biological function, as mentioned above. These two types were also underrepresented as compared to others in overall neighboring codon pairs (Figure 2G). The main types of avoided codon pairs are quite conservative across different domains of life [7,18,20].

It has been reported that translational efficiency can be significantly influenced by transforming synonymous codons and even more validly by codon pairs [8,21]. Thus, the large-scale identification of high-frequency, preferred and avoided codon pairs in *M. amblycephala* (Supplementary file 2), could be used as a reference in design and optimization of exogenous transgenes.

### 2.3. Position-Dependent Codon Usage Bias in M. amblycephala

**Figure 3:**
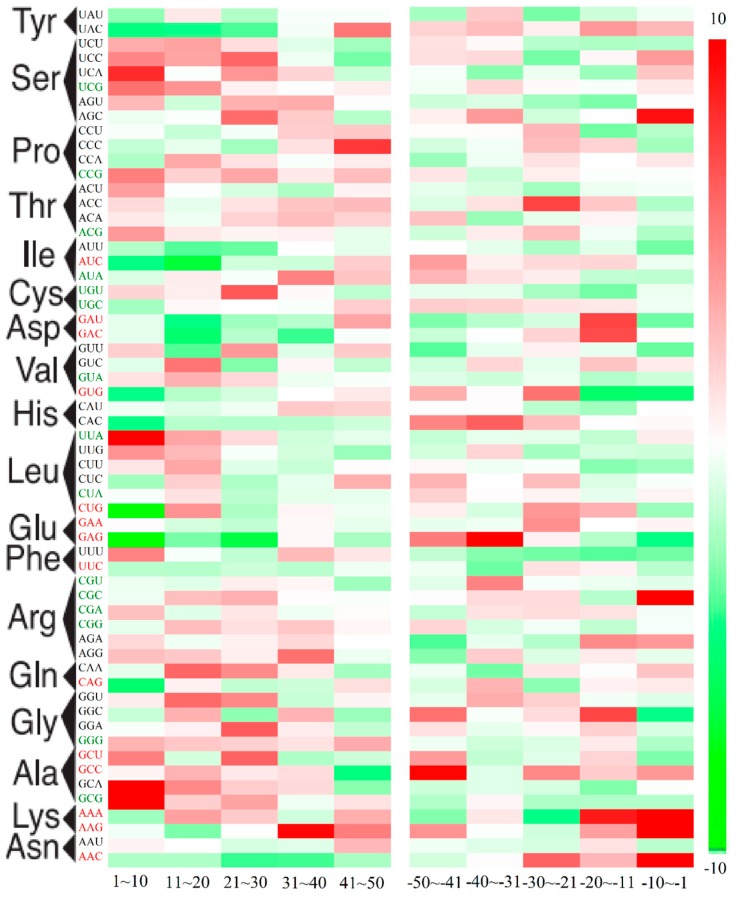
Position-dependent codon usage bias in *M. amblycephala*. KLD value for each codon in *M. amblycephala* is depicted according to the scaled color bar on the right as a function of position: 5 bins following the start codon and 5 bins before stop codons. Rare codons and abundant codons are marked green and red, respectively.

It is widely reported that codon usage bias is not uniform with regard to the position within genes [22,23]. In *M. amblycephala*, the visual analysis of KLD (Kullback–Leibler divergence) values for each codon at the 5ʹ end and 3ʹ end reveals position-dependent heterogeneity (Figure 3). The majority of rare codons were enriched at the beginning of genes and abundant codons were the reverse. Meanwhile, the first 10 codons following the AUG had a preferential selection with A or U at the third position (AU_3_) (Figure S1A). The phenomena could be explained by suppressing mRNA structure and reducing folding propensity for efficient translation initiation [23]. Altogether, the higher values of KLD than a null model indicates an unusual codon usage *N*- or *C*-terminal regions (Figure 3, Figure S1B).

**Figure 4:**
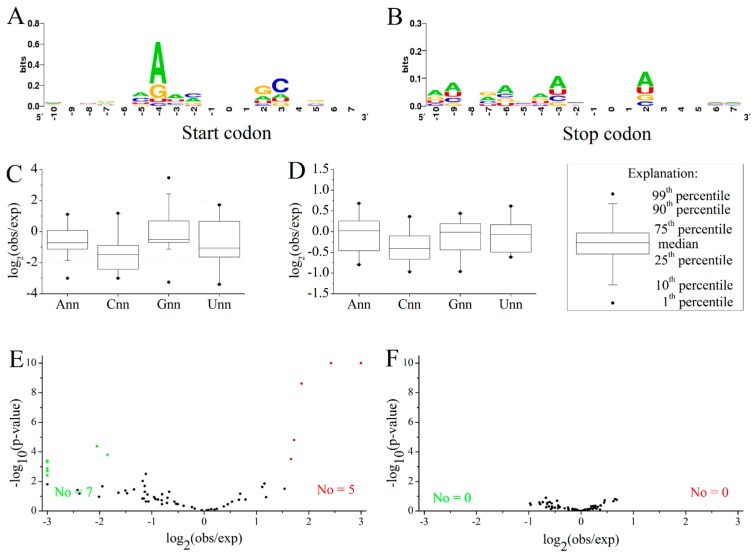
Logo analyses of start and stop codon contexts and the codon bias following AUG in *M. amblycephala*. (**A**,**B**) Logo analyses of 18 nucleotides around the start codon (**A**) and stop codons (**B**). The vertical axis represents the conservation at a certain position (measured in bits). The horizontal axis represents the nucleotide position around the start codon or stop codons. For mapping convenience, the start and stop codons were removed from the resultant map; (**C**,**D**) Distribution of the ratio of observed frequency to expected frequency in different types of codons following the start codon AUG (**C**) and non-start internal codon AUG (**D**); The X axis shows the types of codons, where n represent A, G, C or U; (**E**,**F**) The relationship between observed frequency and expected frequency of 61 codons following the start codon AUG (**E**) and non-start internal codon AUG (**F**). The red, green and black spots represent the preferred codons, avoided codons and unbiased codons, respectively. The lowest *p*-value was set to 1 × 10^−10^.

Moreover, the heterogeneity close to the 5ʹ-region and 3ʹ-region in *M. amblycephala* may shape some preferential flanking sequence characters around start and stop codons (Figure 4A,B). Further analysis showed the preferred nucleotide “G” (Figure 4C) following the start codon AUG, together with “A” just preceding three positions of the start codon AUG, in accordance with the Kozak sequence for identification of the translation start site [24]. The preferential motif would be of species-specific significance in enhancing start codon recognition and translation efficiency [25], which was verified in *Danio rerio* [26]. Meanwhile, 5 preferred and 7 avoided codons were observed for the codon following the start codon AUG (Figure 4E). In contrast, there was no bias observed in the nucleotides or codons following internal AUG codons of genes (Figure 4D,F). This phenomenon confirmed that the bias was related to position-dependent rather than the bias codon following AUG itself. As for the termination codons, a certain relationship with stop codons bias was presented (Figure S1C–E). The maintained bias in stop codon contexts may promote RF (polypeptide release factor) bond efficiency with mRNA to affect translation termination [27,28,29,30].

### 2.4. GC_3_ Bias in M. amblycephala

**Figure 5:**
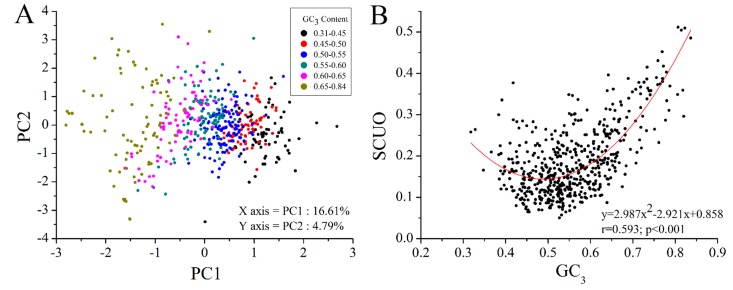
Correlation between GC_3_ content and codon usage bias (RSCU and SCUO) in *M. amblycephala*. (**A**) The PCA analysis of RSCU of 59 codons from 646 ORFs on the primary and secondary axes (accounting for 16.61% and 4.79% of the total variations, respectively) and demonstration by 6 GC_3_ levels; (**B**) SCUO *versus* GC_3_ plot with polynomial fitting.

GC_3_ content varied across *M. amblycephala* transcripts, and its distribution showed a predominantly unimodal type (Figure S2A), which was similar to the earlier observation on other cold-blooded animals [31]. All *M. amblycephala* 646 ORF sequences were performed using PCA based on RSCU to measure the codon usage bias among genes (Figure 5A). Transcripts with different GC_3_ contents could be separated mainly along the first axis, although the percentage of contribution of the axes is somewhat low. These similar correlations have also been reported in some plants [7,32]. SCUO, another index to measure codon usage bias among genes, showed a strong “U” nonlinear correlation with GC_3_ (Figure 5B), similar to the situation in unicellular, human and mouse genomes [33,34]. Above all, codon usage bias showed the pronounced differences across *M. amblycephala* transcripts and had some correlation with GC_3_ content.

**Figure 6:**
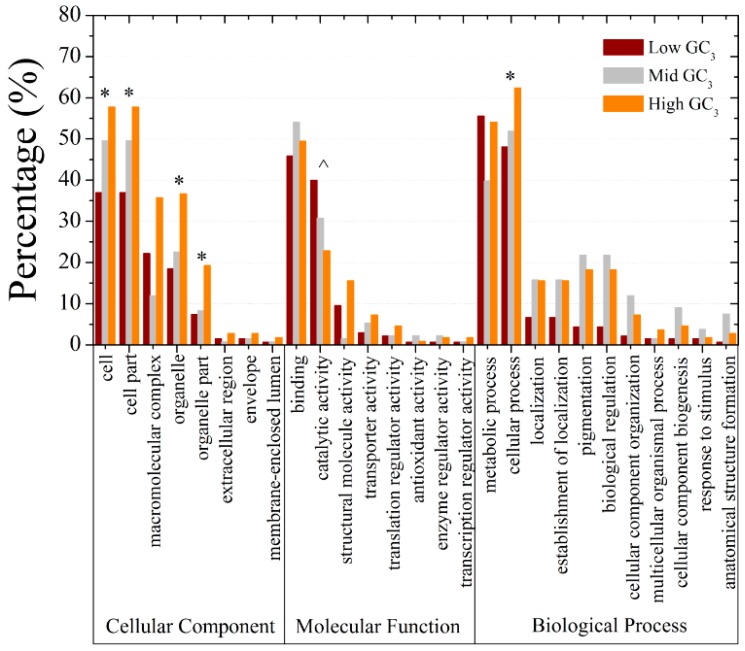
Gene ontology (GO) classifications for three GC_3_ levels in *M. amblycephala*. * indicates, in a particular GO category at the 5% level, a significantly higher percentage of genes in high GC_3_ groups than low GC_3_ groups, while the percentage of mid GC_3_ groups is intermediate; ^ represents an opposite situation.

For a better understanding of the influence of GC_3_ on gene properties in *M. amblycephala*, all the ORFs were almost equally separated into three groups according to GC_3_ value, respectively containing 215, 216 and 215 sequences, for gene ontology (GO) classification analysis. Six GO categories with significant difference among three groups were observed (Figure 6). Five out of six categories, the exception being the “catalytic activity” category, showed positive correlation between gene representation and GC_3_ value. It was further found that GC_3_-rich genes tend to be more enriched in dinucleotide CG (Figure S2B,C), indicating more targets to be methylated [35] for fine-tuning of transcriptional regulation [36]. In contrast, there is no significant difference in the relative abundance of trinucleotide CWG, where W stands for A or T, between the two classes of genes (Figure S2D). The observations support the earlier suggestion that CG and CWG methylation may serve different biological functions [37] in GC_3_-rich genes from in GC_3_-poor genes. The above revealed that GC_3_ bias may be a major factor in driving codon usage bias among genes and relates to gene function and methylation regulation in *M. amblycephala*.

### 2.5. Codon Usage Patterns across the Vertebrates

For comparative analysis on codon usage patterns across the vertebrates, the annotation data of 22 vertebrates, consisting of 8 mammals, 4 birds, one reptile, one amphibian and 8 fishes, were downloaded from ensemble database in addition to the data of *M. amblycephala*.

After filtering thousands of full-length ORFs (16,353 to 104,763), millions of synonymous codons (1,099,276 to 32,166,640) were obtained, and meanwhile, the corresponding GC_1_, GC_2_ and GC_3_ were calculated (Supplementary file 2). In all 23 species (including *M. amblycephala*), GC_1_ and GC_2_ were much more conserved than GC_3_ in the wobble position. GC_1_ and GC_3_ were both much larger than GC_2_, with difference value from 0.125 (*Pelodiscus sinensis*) to 0.153 (*Gadus morhua*), from 0.071 (*Xenopus tropicalis*) to 0.343 (*G. morhua*), respectively. GC_3_ was higher than GC_1_ in fishes and mammals, but was lower in amphibian, reptile and birds except *Taeniopygia guttata*. On the whole, there exists some pressure to select G/C in position 1, T/A in position 2, with significant wide variation in position 3 in vertebrates.

**Figure 7:**
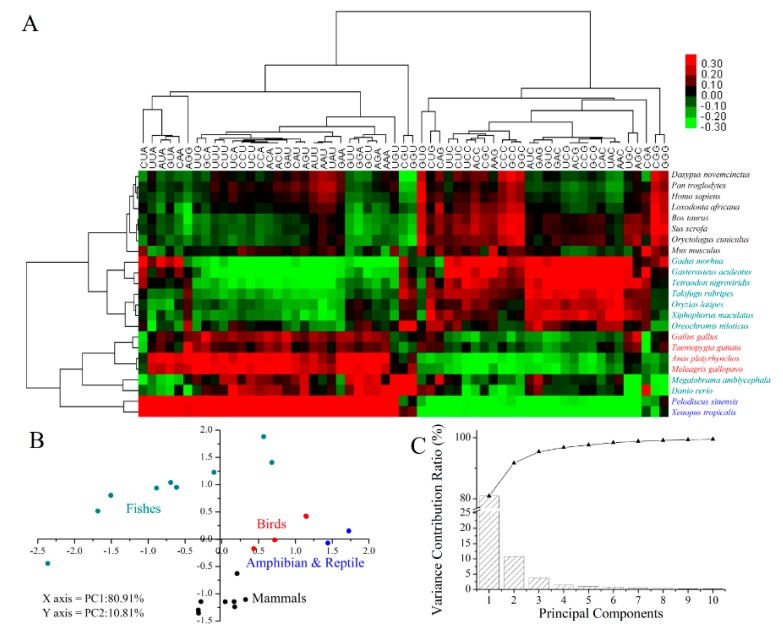
Heat map and PCA analysis of RSCU of synonymous codons from 23 vertebrate species. (**A**) Heat map of adjusted RSCU of 59 codons from 23 species using Euclidean distance and average linkage clustering module; (**B**) The PCA analysis of RSCU of 59 codons from 23 species on the primary and secondary axes (accounting for 80.91% and 10.81% of the total variation, respectively). Dark cyan, blue, red, black represent the fishes, reptile together with amphibian, birds and mammals, respectively; (**C**) Variance contribution ratio (bar) and accumulated variance contribution ratio (triangular plot) of the first ten principal components (*X*-axis) of (**B**).

A heat map via bi-clustering was used to describe the variations of codon usage bias among 23 vertebrate species based on the RSCU of all 59 synonymous codons (Figure 7A). Mammals and most fishes were clustered in a group, while birds, reptile, amphibian and two fishes (*M. amblycephala* and *D. rerio*) were clustered in another. However, the PCA (principal component analysis) test separated these four vertebrate taxonomic groups with the first two principal components (two axes), which account for 80.91% and 10.81% of variations, respectively. The fish group is more scattered than other groups, implying that this group contains much higher variations (Figure 7B,C).

The evolution among vertebrates at the codon-pair level was also evaluated and compared based on the adjusted residual values of codon pair frequencies. The residual values signify the Chi-square test association between the two codons of each pair [38]. The result showed that there remained respective distinctions among fishes, amphibian together with reptile, birds and mammals (Figure S3A–D). The vertebrates showed the uniform feature of higher frequency codon contexts localized diagonally from left top to right bottom, marked by two parallel lines and discrepant bias at NNN–GNN codon contexts (Figure S3A–D), when compared with *Escherichia coli* [20] and insects [13]. Then, the clustering analysis of 23 vertebrate species was performed based on the variation of codon contexts. Though *M. amblycephala* was not well-grouped in the clustering tree, the other fishes, amphibian, reptile, birds and mammals were well-clustered and showed a good correspondence with the known phylogeny of the vertebrate species (Figure S3E, see ToL homepage, http://tolweb.org/tree/phylogeny.html). The fishes cannot be grouped together by RSCU and codon context clustering, which may be due to the complexity of their evolutionary status and their prodigious variation at the codon level compared with other higher vertebrates. The quantity of sequences of *M. amblycephala* used for analysis being less than other samples could be another reason. In the present study, only *M. amblycephala* data is from transcriptome, containing much less sequences than the whole genomes of other 22 species. For the test using the whole trancriptome data, including the incomplete genes, the clustering tree produced good phylogenetic relation among 23 species (Figure S3F). Thus, if the whole genome sequence were available for this analysis, the results could be much clearer.

Codon usage patterns might be formed across hundreds of millions of years as a result of mutation bias, natural selection and genetic drift, as has been observed in yeasts, plants and vertebrates [7,9,39]. To evaluate the evolutionary relationships of vertebrates, the codon context showed more conservation than RSCU, especially in widely divergent species, but relied on a great quantity of sequencing data. Of course, with the development of an evaluation indicator and greater availability of genome sequencing data, such analysis could improve our understanding of codon evolution biology.

## 3. Experimental Section

### 3.1. Sequence Data Collection, Filtering and Mining

Two data sources were applied for this study. Firstly, RNA-Seq data of *M. amblycephala* was downloaded from the NCBI SRA (Sequence Read Archive) database (http://www.ncbi.nlm.nih.gov/Traces/sra/, Accession No.: SRA045792), and was assembled and annotated as in our previous work [4]. Secondly, the protein-coding sequences (*_cds.fa.gz) of 22 published vertebrate genomes were downloaded from ensemble database (http://asia.ensembl.org/).

Open reading frames (ORFs) were determined based on those of similar sequences or predicted by CLC Genomics Workbench v6.5.1 (http://www.clcbio.com/). Then for each species, the full lengths of coding sequences were identified, beginning with an AUG start codon, ending with UAA, UAG or UGA stop codon. From these low-quality sequences, sequences of a length no more than 300 bp and those containing uncertain nucleotides or an internal stop codon were excluded. An additional filtering step for *M. amblycephala* were then used to remove those low-quality sequences which were obviously incomplete or too long (less than 95% or over 105% when compared to the length of top hit homologous sequences from other fishes using BLASTx with an *e*-value cutoff of 10^−5^). All the above procedures were performed with Microsoft Excel 2010 and C programs written in-house.

The first 5 bins (each containing 10 codons, excluding the start codon) and the last 5 bins (excluding the stop codon) in each gene were obtained and mixed together respectively by appointed positions. Then the new rearranged sequences were stored in Fasta format and named as bin_1 to bin_5 and bin_−5 to bin_−1, respectively in the dataset. All the above procedures were performed with Microsoft Excel 2010 and C programs written in-house.

### 3.2. Indices of Codon Usage

GC_i_ is defined as the fraction of cytosines (C) and guanines (G) in the “I” position of the codon: GC_i_ = 3(C_i_ + G_i_)/L for the ORF of length L, and the same definition also used for AU_i_. The indexes above were calculated by Microsoft Excel 2010.

RSCU (Relative synonymous codon usage) is calculated according to the formula described in Sharp and Li [40]. Codons having an RSCU over 1.0 means a high frequency, and the larger the number, the more significant the bias is, while numbers below 1.0 indicate the opposite. The index was calculated with codonW 1.4.2 (http://codonw.sourceforge.net). SCUO (Synonymous codon usage order) was developed based on “Shannon Information Theory” [41] and varied from 0 (no bias) to 1 (most bias). It is calculated using “CodonO” software [42] as 1 minus the ratio of expected to observed entropy, where the expected value of entropy assumes random usage of all synonymous codons of a given amino acid.

### 3.3. Null Models

Two null models were used in the study. SSC (shuffled synonymous codons) means that we preserved the amino acid sequences by shuffling only synonymous codons. SC (shuffled codons) means that codons were randomly permuted.

### 3.4. Identification of High-Frequency, Preferred and Avoided Codons

High-frequency codons are defined as codons with RSCU over 1.5, or those having a relative frequency above 60% of the synonymous codon for the corresponding amino acids [7,43].

The expected frequency is the ratio of total occurrence of a certain codon to the total occurrence of all 61 codons (excluding the stop codons and the AUG when serving as the start codon), calculating from the ORFs (excluding the first and the last codons). The observed frequency is the ratio of actual occurrence of a certain codon in a certain position of all ORFs to total occurrence of all 61 codons in that position. The frequencies of the codons following the start codon AUG and those following non-start internal AUG codon were also calculated. Frequencies with *p*-values less than 0.01 were considered statistically significant, and the ratio of observed frequency to expected frequency (log_2_), with a ratio cutoff of ±1.5 (3-fold changes), was the standard used to identify the preferred or avoided codons. *p*-value was calculated following the formula described in Audic and Claverie [44] via the PERL program [45].

### 3.5. Identification of High-Frequency, Preferred and Avoided Codon Pairs

High-frequency codon pairs are defined as codons with RSCPU (relative synonymous codon pair usage) over 1.5, or those having a relative frequency above 60% of synonymous codon pairs for the corresponding amino acid pairs. The RSCPU is the observed frequency of codon pairs divided by the expected frequency, which in turn is the total number of amino acid pairs divided by total number of codon pairs that code the same amino acid pair. The sum of numbers of all the codon pairs that code for the same amino acid pair was divided by the product of codon degeneracies of both the amino acids to obtain the expected values. Identification of the high-frequency codon pairs was performed using Microsoft Excel 2010 and C programs written in-house.

The expected frequency of 3721 (61 × 61) codon pairs (both the neighboring codon pairs and those separated by several intervening codons) is the product of the corresponding expected frequencies of each codon. The observed frequency of codon pairs is the ratio of occurrence of a certain pair to occurrence of all 3721 codon pairs, calculating from full length ORFs excluding the first and stop codons, as mentioned above. The parameters for screening preferred and avoided codon pairs were the same as described above. All procedures were performed using Microsoft Excel 2010, C programs written in-house and the PERL program [45].

### 3.6. Calculation of KLD Value and Logo Analyses

The position-dependent KLD(*k*) that quantifies the deviation of the codon usage in each bin *k* is calculated according to the report [23]:

(1) $$\text{KLD}\left(k\right)=\;\overset{20}{\underset{i\;=\;1}{\sum }}\overset{S_{i}}{\underset{j=1}{\sum }}p_{i,j}\left(k\right)\text{ln}\frac{p_{i,j}\left(k\right)}{q_{i,j}}$$

where *q*_*i*,*j*_ is the frequency of each codon within each set of synonymous codons, *i* = 1…20 indicates the amino acid and *j* = 1…*S_i_*, indexes the synonymous codon (where *S_i_* is number of synonymous codons); *p*_*i*,*j*_(*k*) is the position-dependent codon frequency in each bin *k*. Because of finite size effects, the KLD(*k*) is biased to values larger than 0 even if *p*_*i*,*j*_ and *q*_*i*,*j*_ stem from the same distribution [46]. The bias due to this finite size sampling was estimated using the SSC null model.

The 18 nucleotides (including 9 nucleotides before the start or stop codons, 3 nucleotides of the start or stop codons and 6 nucleotide following the start or stop codons) of each mRNAs were picked out via C programs written in-house and performed using the Web site tool, WebLogo [47]. For mapping convenience, the start and stop codons were removed from the resultant map.

### 3.7. Calculation of Relative Abundance

Relative abundance was calculated according to the report [48], in which the profiles of relative dinucleotide abundance values are equivalent to the “general design” of organisms, and the computational formulae for di- and tri-nucleotide relative abundance values are ρ_CG_ = *f*_CG_/*f*_C_*f*_G_, ρ_CWG_ = *f*_CWG_*f*_C_*f*_W_*f*_G_/*f*_CW_*f*_WG_*f*_CNG_, where W stands for A or U, N stands for A,T,C or G; *f*_x_ denotes the frequency of the nucleotide X, *f*_xy_ the frequency of the dinucleotide XY, *f*_xyz_ the frequency of the trinucleotide XYZ.

### 3.8. Gene Ontology Annotation

Gene Ontology annotation of full length ORFs was performed using Blast2GO (http://www.blast2go.com) [49], and GO classifications were compared among three GC_3_ levels using WEGO (http://wego.genomics.org.cn/cgi-bin/wego/index.pl) [50].

### 3.9. Clustering and Principal Component Analysis (PCA)

The RSCU values of codons among the 23 species were clustered using a hierarchical clustering method (average linkage) implemented in Cluster 3.0 software [51]. The rank order correlation-based similarity matrix of the RSCU values was used to determine clusters among codons (columns) and species (rows). The clusters were viewed by the TreeView program (version 1.60, University of California, Berkeley, CA, USA) (http://www.eisenlab.org/eisen/).

PCA of these 23 vertebrates were performed based on the RSCU of 59 synonymous codons, while the variance contribution ratio and accumulated variance contribution ratio were calculated using SPSS (version 19.0) and drawn with OriginLab Origin (version 8.0, Microcal Software Inc., Northampton, MA, USA). All the full length ORFs with the RSCU value of 59 synonymous codons (1066 spots) were reduced from 59 dimensions (59 codons) into two principal components, using the same procedure.

### 3.10. Codon Context Analysis

The residual values of each codon pair were quantified from the coding sequences of each genome or RNA-Seq data by the Anaconda program [20]. The cluster trees were generated by the same program after comparison among the codon context patterns of each species.

## 4. Conclusions

A comprehensive analysis of usage bias of genetic codons and codon pairs in *M. amblycephala* was performed. Underrepresentation of codons NUA and NCG was observed, which may have the functions for controlling protein production and avoiding the mutation caused by DNA methylation, respectively. The majority of amino acid pairs had obvious bias for synonymous codon pairs. The prominent biases on neighboring codon pairs are possibly significant in regulating protein synthesis rate. Position-dependent heterogeneity of codon usage was apparently close to start and stop codons, and flanking sequence feature could contribute to efficient translation initiation and termination. Codon usage bias (RSCU and SCUO) may be driven by GC_3_, and six GO categories and methylation regulation were both influenced by GC_3_. Among vertebrate species, GC content, RSCU and codon context pattern all had species specificities and the codon context was relatively better for vertebrate evolution estimation.

This study provides insight into fish codon biology, vertebrate evolution and fish breeding projects, such as codon optimization and transgene fish establishment, and the extensive application of RNA-Seq data.

## Supplementary Materials

Supplementary materials can be found at http://www.mdpi.com/1422-0067/16/06/11996/s1.

## Abbreviations

AU_3_: AU content at 3rd nucleotide in codon
C: Cytosine
G: Guanine
GC_1_: GC content of 1st nucleotide in codon
GC_2_: GC content of 2nd nucleotide in codon
GC_3_: GC content of 3rd nucleotide in codon
GO: Gene ontology
KLD: Kullback–Leibler divergence
ORF: Open reading frame
PCA: Principal component analysis
RSCU: Relative synonymous codon usage
RSCPU: Relative synonymous codon pair usage
SSC: shuffled synonymous codons
SC: shuffled codons
SCUO: Synonymous codon usage order
ToL: The Tree of Life Web Project
