# Supplementary material for: A Comprehensive Analysis of Codon Usage Patterns in Blunt Snout Bream (Megalobrama amblycephala) Based on RNA-Seq Data
Source: Int J Mol Sci. 2015 May 26;16(6):11996–2013. doi: 10.3390/ijms160611996 (PMC4490425; doi:10.3390/ijms160611996)
Supplement: Supplementary file 1 [file ijms-16-11996-s001.zip › ijms-82128-Supplementary Information/ijms-82128-Supplementary file 2.pdf]

Supplementary Information

Table S1. Codon usage table in *Megalobrama amblycephala.*

AA	Codon	NO. [a]	1/1k [b]	RSCU [c]	AA	Codon	NO. [a]	1/1k [b]	RSCU [c]	AA	Codon	NO. [a]	1/1k [b]	RSCU [c]	AA	Codon	NO. [a]	1/1k [b]	RSCU [c]
Phe	UUU	2444	17.71	0.91	Ser	UCU	2014	14.59	1.24	Tyr	UAU	1707	12.37	0.85	Cys	UGU	1378	9.99	1.09
	UUC	2918	21.14	1.09		UCC	1812	13.13	1.11		UAC	2328	16.87	1.15		UGC	1157	8.38	0.91
Leu	UUA	926	6.71	0.46		UCA	1611	11.67	0.99	Ter	UAA	240	1.74	1.11	Ter	UGA	278	2.01	1.29
	UUG	1719	12.46	0.85		UCG	610	4.42	0.37		UAG	128	0.93	0.59	Trp	UGG	1505	10.91	1.00
	CUU	1885	13.66	0.93	Pro	CCU	1790	12.97	1.08	His	CAU	1477	10.70	0.89	Arg	CGU	1186	8.59	0.86
	CUC	2241	16.24	1.11		CCC	1630	11.81	1.07		CAC	1845	13.37	1.11		CGC	1285	9.31	0.93
	CUA	774	5.61	0.38		CCA	1877	13.60	1.24	Gln	CAA	1565	11.34	0.52		CGA	892	6.46	0.65
	CUG	4585	33.22	2.27		CCG	779	5.64	0.51		CAG	4495	32.57	1.48		CGG	779	5.64	0.56
Ile	AUU	2347	17.01	1.02	Thr	ACU	1871	13.56	1.02	Asn	AAU	2110	15.29	0.81	Ser	AGU	1637	11.86	1.01
	AUC	3408	24.70	1.48		ACC	2214	16.04	1.21		AAC	3122	22.62	1.19		AGC	2084	15.10	1.28
	AUA	1139	8.25	0.50		ACA	2286	16.56	1.25	Lys	AAA	4778	34.62	0.91	Arg	AGA	2330	16.88	1.69
Met	AUG	4010	29.06	1.00		ACG	964	6.99	0.53		AAG	5755	41.70	1.09		AGG	1810	13.12	1.31
Val	GUU	2117	15.34	0.95	Ala	GCU	3101	22.47	1.30	Asp	GAU	3628	26.29	0.97	Gly	GGU	2033	14.73	0.96
	GUC	2193	15.89	0.99		GCC	3017	21.86	1.27		GAC	3864	28.00	1.03		GGC	2267	16.43	1.07
	GUA	957	6.93	0.43		GCA	2344	16.99	0.98	Glu	GAA	3592	26.03	0.72		GGA	2887	20.92	1.36
	GUG	3611	26.17	1.63		GCG	1063	7.70	0.45		GAG	6326	45.84	1.28		GGG	1277	9.25	0.60

[a] the number of codons in this set; [b] Codon frequency normalized per 1000 bases; [c] RSCU, relative synonymous codon usage.

Table S2. Basic information of ORFs of 22 vertebrates.

Species	Classification	No. Raw [a]	No. Filter [b]	GC_1 [c]	GC_2 [d]	GC_3 [e]	NCG/NCC [f]	L_sym [g]	GC_{3s} [h]
Anas platyrhynchos	bird	16,353	2378	0.527	0.401	0.508	0.352	1,395,584	0.49
Bos taurus	mammal	22,118	15,384	0.568	0.425	0.613	0.337	7,865,724	0.599
Danio rerio	fish	43,153	27,662	0.541	0.412	0.546	0.437	14,664,501	0.529
Dasypus novemcinctus	mammal	26,551	14,778	0.56	0.423	0.578	0.327	7,762,039	0.563
Gadus morhua	fish	22,100	2821	0.566	0.412	0.756	0.44	1,099,276	0.746
Gallus gallus	bird	16,354	11,726	0.544	0.411	0.537	0.375	6,787,730	0.52
Gasterosteus aculeatus	fish	27,576	9239	0.565	0.417	0.708	0.499	4,414,263	0.697
Homo sapiens	mammal	104,763	63,459	0.564	0.427	0.579	0.288	32,166,640	0.565
Loxodonta africana	mammal	25,635	8608	0.563	0.421	0.59	0.283	4,384,775	0.575
Meleagris gallopavo	bird	16,496	3656	0.532	0.401	0.509	0.313	1,992,550	0.491
Mus musculus	mammal	52,165	37,773	0.558	0.423	0.579	0.281	19,974,206	0.564
Oreochromis niloticus	fish	26,763	15,238	0.551	0.418	0.6	0.366	8,651,545	0.585
Oryctolagus cuniculus	mammal	20,588	12,623	0.563	0.424	0.608	0.362	6,967,361	0.595
Oryzias latipes	fish	24,674	7939	0.553	0.414	0.634	0.416	3,370,200	0.619
Pan troglodytes	mammal	19,907	15,885	0.559	0.423	0.568	0.296	8,437,496	0.553
Pelodiscus sinensis	reptile	20,669	10,977	0.527	0.402	0.492	0.312	5,789,771	0.473
Sus scrofa	mammal	25,882	13,504	0.57	0.428	0.612	0.328	6,290,142	0.599
Taeniopygia guttata	bird	18,204	3248	0.539	0.405	0.556	0.311	1,656,264	0.54
Takifugu rubripes	fish	47,841	10,618	0.559	0.419	0.652	0.427	6,317,041	0.639
Tetraodon nigroviridis	fish	23,118	7496	0.565	0.423	0.692	0.451	3,580,415	0.68
Xenopus tropicalis	amphibian	22,718	9986	0.526	0.401	0.472	0.3	4,973,237	0.453
Xiphophorus maculatus	fish	20,454	12,805	0.554	0.416	0.638	0.478	6,910,273	0.625

[a] the numbers of original sequences from ORF annotation and protein annotation; [b] the number of full length coding sequences after filtering; [c] the GC content of 1st codon; [d] the GC content of 2nd codon; [e] the GC content of 3rd codon; [f] the ratio of CG-end codons and CC-end codons; [g] the number of synonymous codons; [h] the GC content of 3rd synonymous codon.

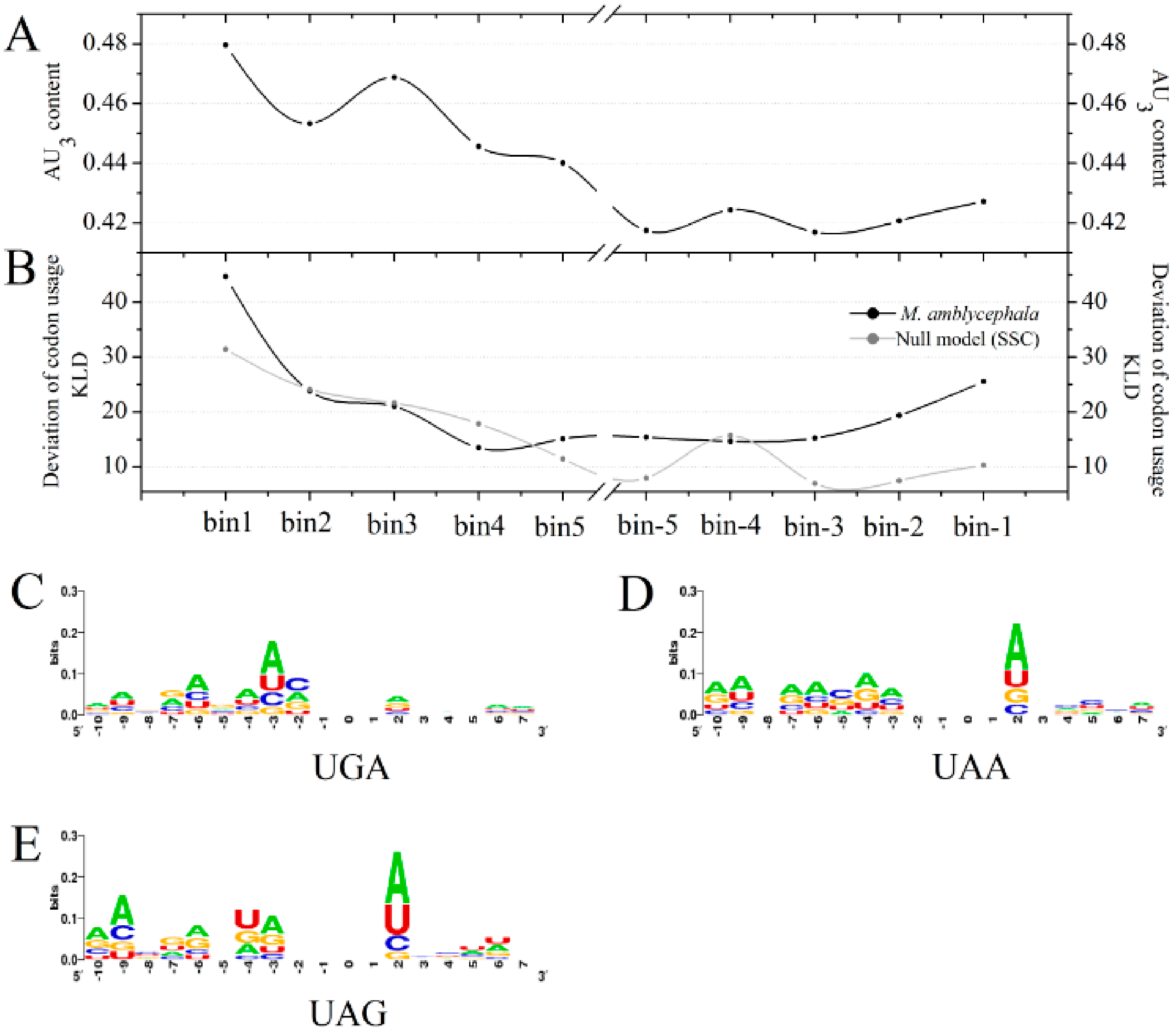

Figure S1. AU3 value and KLD value across dependent positions and logo analyses of three stop codon contexts. (**A**,**B**) AU3 value (**A**) and KLD value (**B**) are demonstrated as a function of position: 5 bins following the start codon and 5 bins before stop codons; (**C**–**E**) Logo analyses of 18 nucleotides around the UGA (**C**), UAA (**D**), UAG (**E**). For mapping convenience, the start and stop codons were removed from the resultant maps.

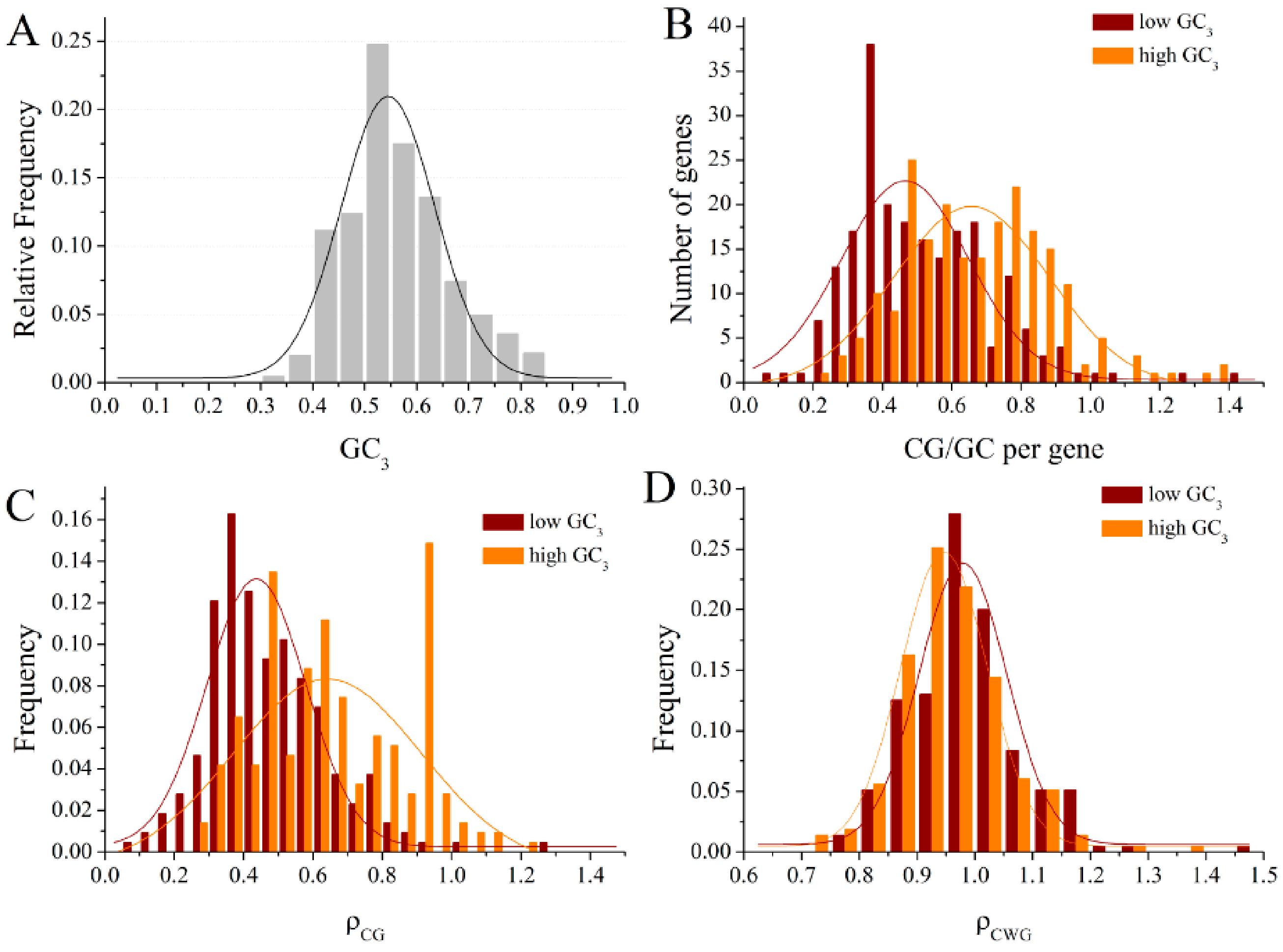

Figure S2. GC_3 distribution in *M. amblycephala* (**A**) and distribution of ratio of dinucleotide frequencies CG to GC (**B**), relative abundance of CG (**C**) and CWG (**D**) in high and low GC_3 groups.

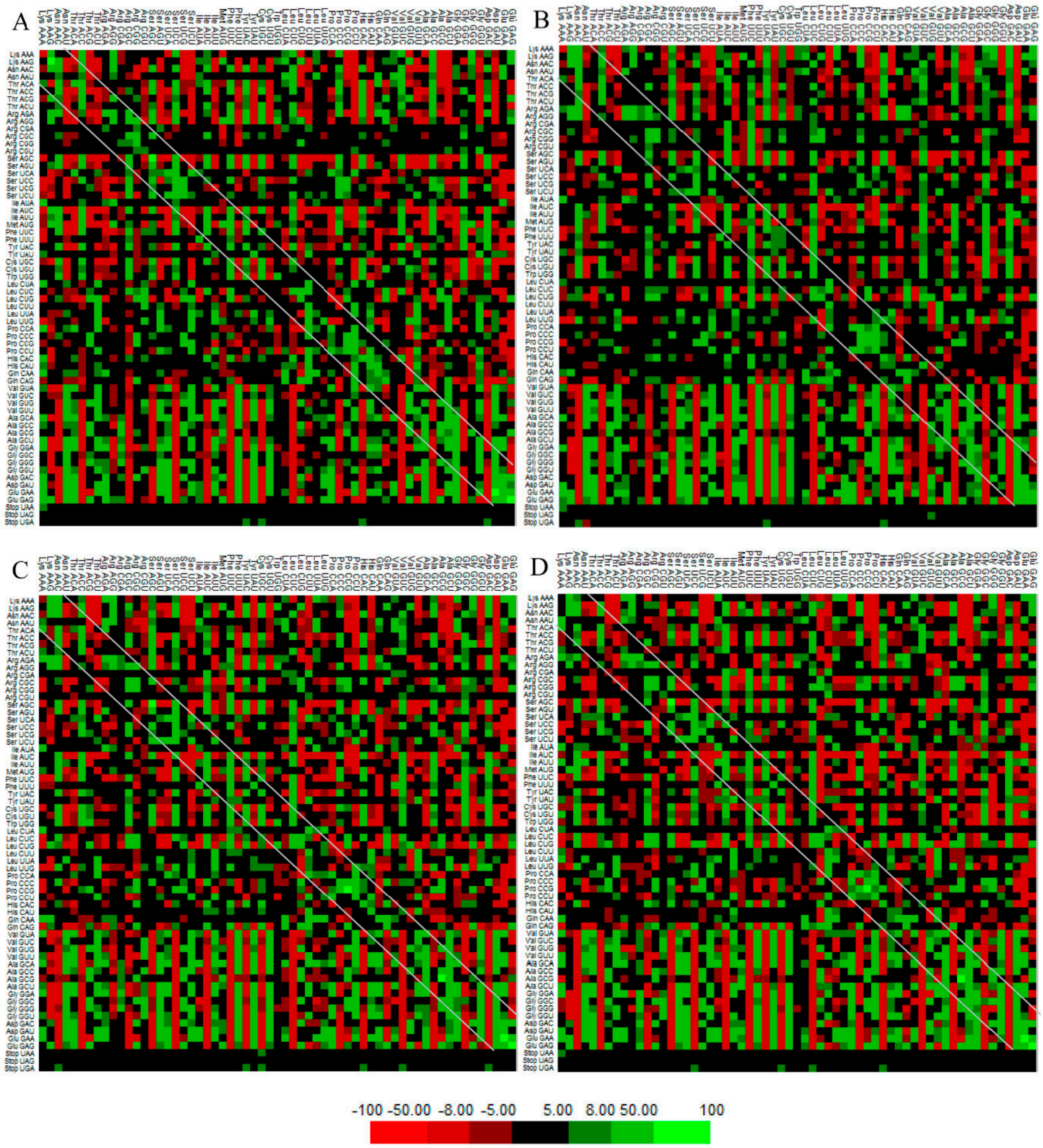

Figure S3. *Cont.*

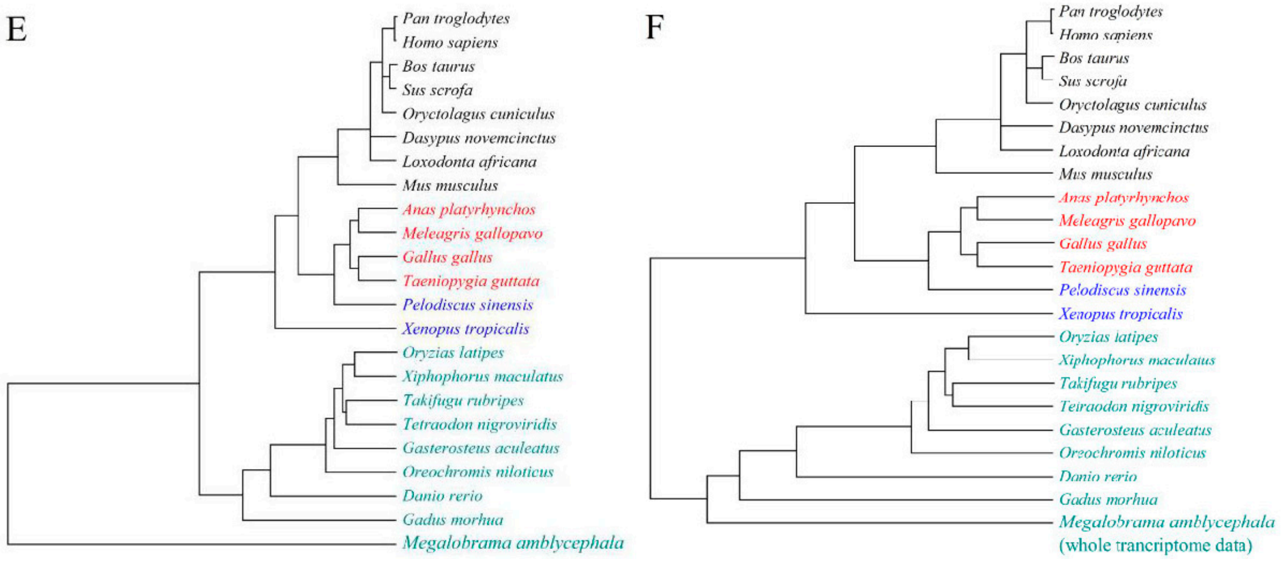

Figure S3. Patterns of codon context variations among 4 four vertebrate taxonomic groups and cluster analysis among 23 vertebrate species. (**A**–**D**) The cluster pattern is based on average matrix of residuals of each codon context among fishes (**A**), reptile together with amphibian (**B**), birds (**C**) and mammals (**D**), respectively. The 5′ codons are in columns and the 3′ codons are in rows. The green color represents highest number of the contexts and red color represents the lowest number of contexts as shown at the bottom of the **A**–**D**; (**E**,**F**) Two cluster trees depicting codon context variations among 23 vertebrate species by using full-length ORF sequences of *M. amblycephala* (**E**) and whole trancriptome coding sequences of *M. amblycephala* (**F**). Dark cyan, blue, red, black represent the fishes, reptile together with amphibian, birds and mammals, respectively.
